# Pharmacist-led medication reviews in primary healthcare for adult community-dwelling patients – a descriptive study charting a new target group

**DOI:** 10.1186/s12875-022-01849-x

**Published:** 2022-09-16

**Authors:** Katarina Wickman, Annika Dobszai, Sara Modig, Beata Borgström Bolmsjö, Gabriella Caleres, Cecilia Lenander

**Affiliations:** 1 Department of clinical sciences, Lund University, Malmö, Sweden; 2 Primary Healthcare, Skåne County, Lund, Sweden; 3 Department of Medicines Management and Informatics in Skåne County, Malmö, Sweden

**Keywords:** Drug-related problem, Medication review, Primary healthcare, Pharmacist, clinical, Independent living

## Abstract

**Background:**

Medication treatment can reduce morbidity but can also cause drug-related problems (DRPs). One method to identify and solve DRPs is medication reviews (MRs) that are aimed at increased patient safety and quality in drug treatment. In Skåne county, Sweden, a well-established multi-professional model for MRs in nursing homes is practiced. However, a demand for MRs regarding community-dwelling patients has emerged. These patients may be extra vulnerable since they have less supervision from healthcare personnel.

AIM: To describe the community-dwelling patients in primary healthcare considered in need of an MR, as well as the outcomes of these pharmacist-led MRs.

**Methods:**

Personnel from 14 primary healthcare centers selected patients for the MRs. Based on electronic medical records, the symptom assessment tool PHASE-20 (PHArmacotherapeutical Symptom Evaluation 20 questions) and medication lists, pharmacists conducted MRs and communicated adjustment suggestions via the medical record to the general practitioners (GPs).

**Results:**

A total of 109 patients were included in the study and 90.8% (*n* = 99) of the patients were exposed to at least one DRP, with an average of 3.9 DRPs per patient. Patients with impaired renal function (glomerular filtration rate, GFR < 45 ml/min) or ≥ 10 medications were exposed to a significantly higher number of DRPs per patient, 5.1 DRP and 5.3 respectively. The most frequent DRP-categories were *Unnecessary drug therapy* and *Adverse drug reaction,* which represented 23.0% respectively 22.9% of the total amount of DRPs.

**Conclusions:**

Our results indicate a prioritized need for MRs for community-dwelling patients, specifically with impaired renal function or polypharmacy.

**Supplementary Information:**

The online version contains supplementary material available at 10.1186/s12875-022-01849-x.

## Background

New medications and treatment guidelines enable care for many diseases but also contribute to polypharmacy regardless of age [1–3]. Increased medication use leads to a higher risk of drug-related problems (DRPs) [4]. A DRP can be defined as an undesirable patient experience that involves drug therapy and one that can potentially interfere with a desired patient outcome [1, 2]. One method to identify and solve DRPs is medication reviews (MRs). The method entails a structured methodical evaluation aiming to optimize medication effect and to prevent adverse events [5, 6]. The Swedish National Board of Health and Welfare mandates MRs for elderly patients with five medications or more when those patients are in need of healthcare [6].

Like many countries, Sweden has an increasing proportion of elderly people in the population. According to Statistics Sweden, 20% of the population in 2020 was 65 years of age or older [7]. Most of these seniors continue to live independently. Nursing homes and other institutional settings are mainly for the frailest elderly with multimorbidity. Consequently, numerous elderly with multimorbidity and polypharmacy will be community-dwelling, with or without municipal healthcare [8].

In Skåne County, in the southern part of Sweden, there is an established model for MRs with multiprofessional teams consisting of clinical pharmacists, general practitioners (GP) in primary healthcare centers (PHCC) and nurses at nursing homes/community [9]. The target group for the MRs has mainly been patients in nursing homes, but to some extent also community-dwelling patients enrolled in municipal healthcare. The MRs are performed using a modified version of the Lund Integrated Medicine Management (LIMM) model adapted for primary healthcare [10, 11].

Over the last few years, the demand for MRs for community-dwelling patients has increased. This population is often responsible for their own medication administration, thus lacking frequent monitoring from professional healthcare regarding, for example, adverse effects. These patients may be particularly vulnerable as they also often suffer from multimorbidity and polypharmacy [12, 13].

A few national and international studies exist regarding MRs in primary healthcare for community-dwelling patients; these show a vast variation in the average number of DRPs between 1.37–7.2 per patient [14–18]. There are studies on MRs conducted in Swedish primary healthcare [19, 20], but the focus has largely been on MRs in nursing homes and more research is required to assess the need and where to focus regarding MRs for patients living independently.

The aim of this study was to describe the group of community-dwelling patients in primary healthcare considered in need of a structured pharmacist-led MR, as well as their outcomes regarding DRPs and involved medications.

## Method

### Study design

This study was a retrospective study with descriptive analysis of data collected from MRs for patients that were living independently.

### Setting and participants

In this study, the analysis was based on data from community-dwelling patients registered to a public primary healthcare center (PHCC) in the southern part of Skåne County, who received an MR conducted by a multi-professional team. The team consisted of a clinical pharmacist from Skåne University Hospital, a GP, and in some cases a nurse from the public PHCC. The GP or nurse at the PHCC identified patients in their daily work for whom they considered an MR was of interest, due to for example suspected DRPs. No further inclusion criteria were required; the patients could be included regardless of the number of medications. Patients living in nursing homes or other institutional settings, younger than 18 years, registered to private PHCCs or with protected identity were excluded. Ethics approval was granted, and informed consent was obtained from all patients included in the study.

### Procedure

Selected patients answered PHASE-20 (PHArmacotherapeutical Symptom Evaluation, 20 questions), an assessment tool that can be used to identify possible drug-related symptoms [21]. The scale ranges from none, small, moderate to severe discomforts during the last 2 weeks. A current medication list was compiled by the patient. A nurse (or a relative) could assist the patient if needed. The documents were sent by mail to the clinical pharmacists who then initiated an MR according to the modified version of LIMM [10, 11] and also confirmed the patient’s medication list with the electronic medical record (EMR). The clinical pharmacist identified potential DRPs based on symptoms, medication use, information from the EMR and laboratory tests according to instructions in internal documents. Renal function, GFR (Glomerular Filtration Rate), was estimated from creatinine and when accessible supplemented with cystatin C. When needed, the clinical pharmacist contacted the patient for additional information. Within 3 weeks of receiving the documents, the pharmacist documented DRPs and subsequent recommendations in the EMR (Table 1). Identified medication discrepancies were also documented in the EMR.

**Table 1:** Interpretation of standard template categories of DRP from the EMR

Categories of DRPs documented in the EMR according to local instructions and standard template	Interpretation
TDM medication	TDM; therapeutic drug monitoring, Drugs requiring therapeutic monitoring
Suitability for elderly	Potentially inappropriate drugs for elderly patients according to The Swedish National Board of Health and Welfare
Drugs not recommended	According to the Regional drug and therapeutics committee
Problems with administration/handling	For example, medication crush, cut, inhalation technique
Drug interactions	C/D drug- drug interactions
Choice of drug/dosage	Dose not adjusted for the patient (i.e. in relation to renal or hepatic function)
Unclear indication/not documented	No information/unclear indication for medication treatment, or weak evidence for specific medication
Additional treatment	Suboptimal treatment
Potential adverse drug reaction	An undesired effect of medication treatment
No identified DRP	

The MR was communicated from the pharmacist to the GP through a written message in the EMR. When the MR was more complex or needed to be discussed, additional discussion by phone or digital meeting was arranged. The GP had the medical responsibility and final decision regarding all proposed recommendations and if these should be implemented. Follow-up took place according to standard care.

### Data collection

Data were collected from MRs processed during the 3rd quarter 2018 – 4th quarter 2020. The research team retained information from MRs documented in the EMR, medication list, PHASE-20 and notes taken from the MR. Data were registered in Microsoft Excel and the analyses were carried out in IBM SPSS version 28.0.

### Data analysis

Descriptive and comparative (Mann-Whitney U test and Chi-square test, respectively) analysis was used to process the information from the MRs including patient data. Number of DRPs per patient was assessed. Since patients under the age of 65 years could be included, Beers criteria or STOP and START were not suitable due to their focus on elderly. DRPs documented in the EMR were therefore recategorized according to Cipolle, Strand, Morley et al. [1, 2] and since previous studies also apply this model. Two pharmacists conducted the recategorization according to the seven classification categories. In case of discrepancy or difficulty in the classification, the pharmacists reached consensus through discussion.

1. Need for additional therapy
2. Unnecessary drug therapy
3. Wrong drug
4. Dose too low
5. Adverse drug reaction
6. Dose too high
7. Adherence problems

## Results

Patients registered to 14 PHCCs in the southern part of Skåne County were selected for MRs. In all, MRs were conducted for 165 patients during 2018–2020 and 109 of these were included in the study. Informed consent to participate in the study was obtained during the second half of 2020. A total of 56 patients were excluded; 18 declined, 15 could not give consent due to cognitive impairment, 11 were deceased, 11 could not be reached and one did not meet the inclusion criteria due to living in a nursing home.

Among the included patients frequent diagnoses documented in the EMR were cardiovascular disease, pain, diabetes, asthma/chronic obstructive pulmonary disease (COPD) and mental illness/psychiatric diagnoses. Baseline patient characteristics are presented in Table 2.

**Table 2:** Baseline characteristics of included patients (*n* = 109)

Patient baseline characteristics	*n* = 109
Age (years), mean (range)	79 (52–98)
Female, n (%)	60 (55.0)
GFR < 45 ml/min, n (%)	28 (25.7)
Number of medications per patient, mean (range)	12 (5–28)
Number of continuous medications, mean (range)	9 (3–20)
Number of medications as needed, mean (range)	3 (0–11)
Number of patients who handle medications without any support from healthcare, n (%)	84 (77.1)
Patients with < 5 continuous medications n (%)	6 (5.5)
Patients with 5 - < 10 continuous medications, n (%)	60 (55.0)
Patients with ≥10 continuous medications, n (%)	43 (39.4)
Service; patient-specific dispensing of medications^a^ n (%)	24 (22.0)

^a^Repackaging of solid oral medications used regularly, into unit-dose bags for each time of administration

**Fig. 1:**
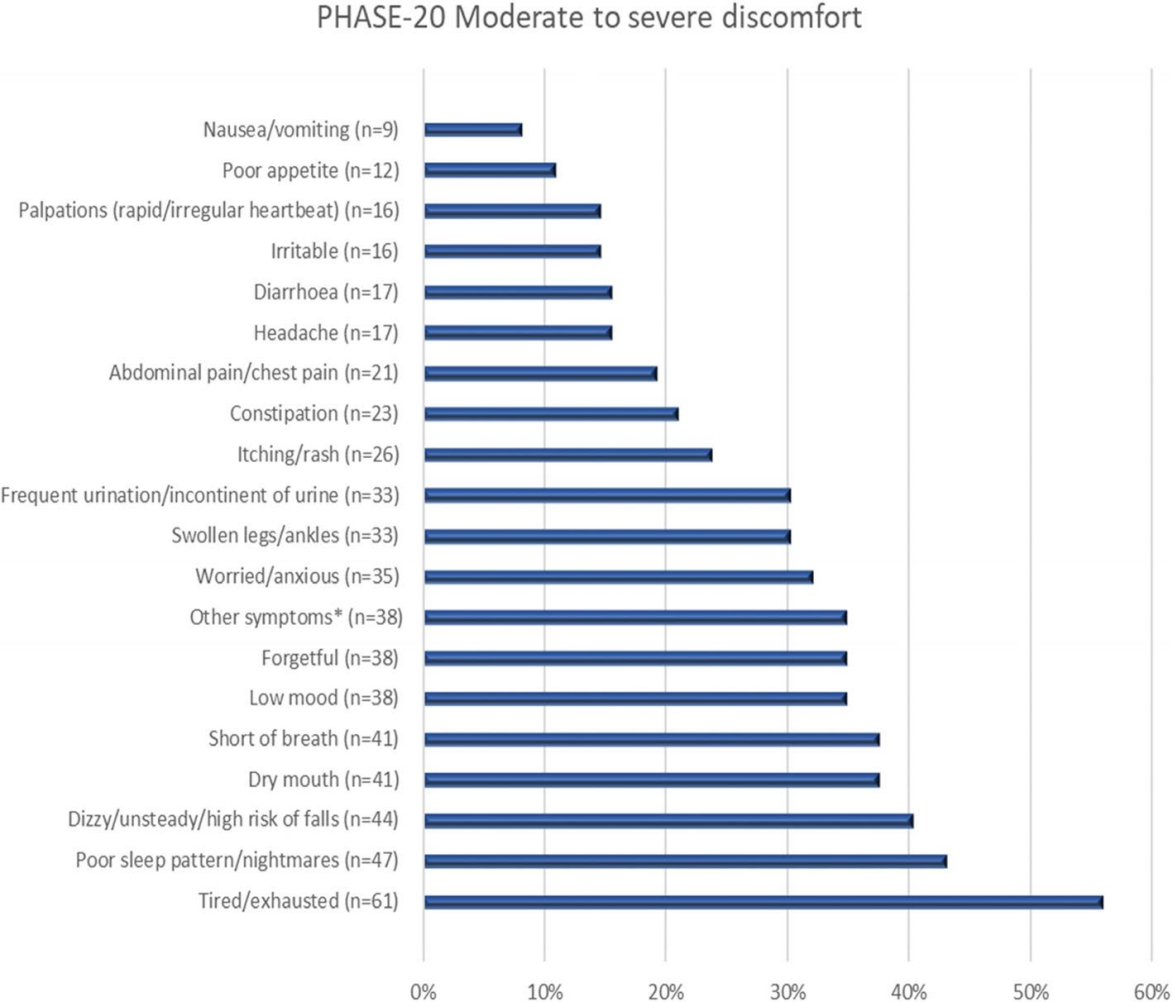
Reported moderate to severe discomfort using PHASE-20 (assessment tool) by the patients (*n* = 109). *The majority of “Other symptoms” concern pain (30 out of 38)

Figure 1 shows the proportion of patients who reported moderate to severe discomfort in each question in the assessment tool, PHASE-20. When summing up pain from the different categories, regardless of the degree of discomfort, a total of 80 (73.4%) patients reported pain.

**Fig. 2:**
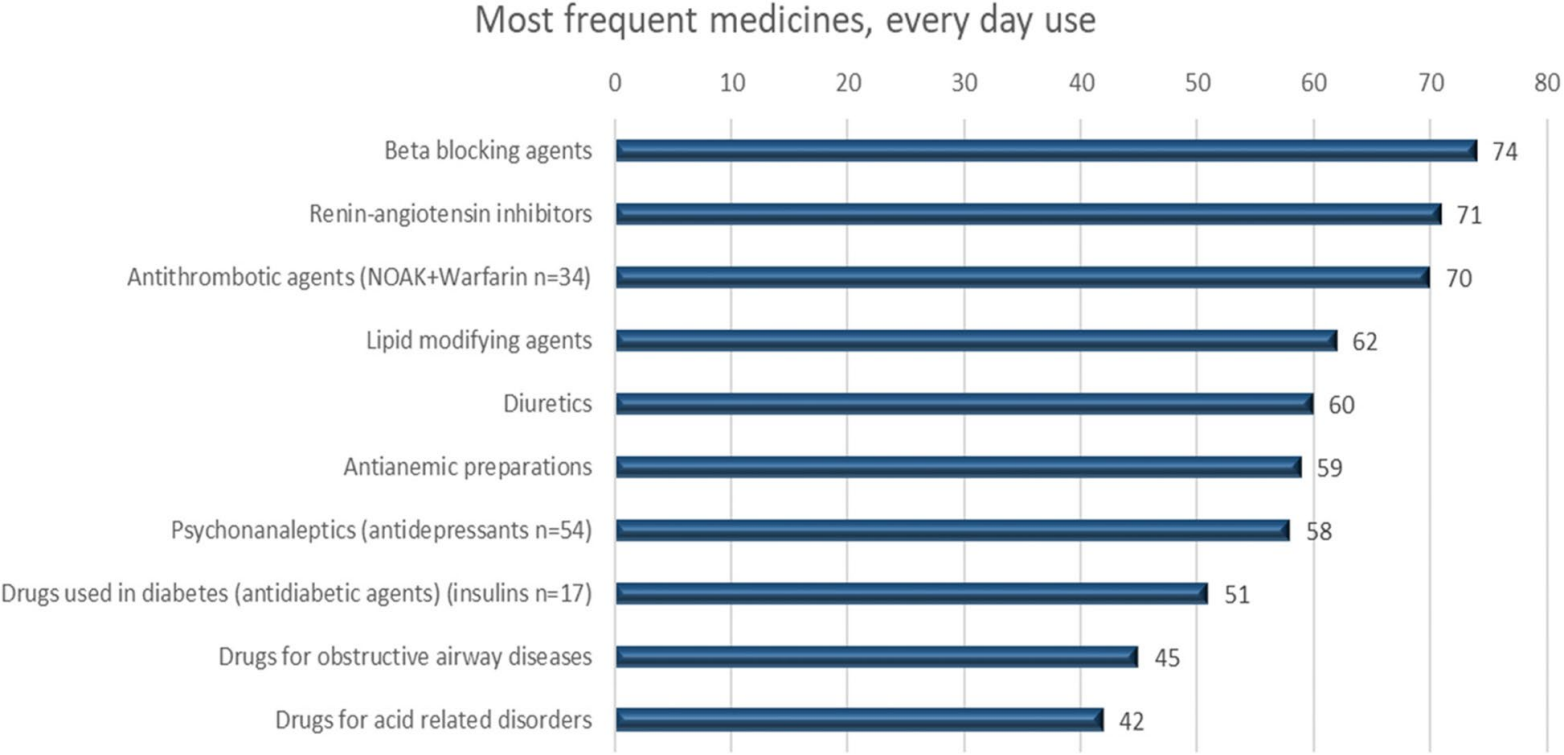
Most frequent medications according to ATC 3rd level (number of patients using, n). One patient can use more than one medication in each category

The most frequent medications overall, categorized according to Anatomical Therapeutic Chemical classification system 3rd level (ATC) [22] in the patient group, were C07; beta-blocking agents, C09; renin angiotensin inhibitors and B01; antithrombotic agents, (Fig. 2). N02; analgesics were used by 71 patients (65.1%) overall (as needed or continuous use) and by 27 patients (24.8%) for continuous use. Regarding treatment with N05; psycholeptics, a total of 27 patients (24.8%) had at least one continuous medication. Of these, 20 patients (18.3%) were found to use N05C; Hypnotics and sedatives every night. In the ATC group N06; psychoanaleptics four patients (3.7%) used memantine or cholinesterase inhibitors.

### Drug-related problems

A total of 420 DRPs were identified by the pharmacists, resulting in a mean of 3.9 DRPs per patient and a median of 4 DRP per patient (range 0–13) (Table 3). Most of the patients (*n* = 99, 90.8%) were exposed to at least one DRP. Patients with age < 75 years were found to have a mean of 2.9 DRPs (median 3) and patients ≥75 years had 4.2 DRPs (median 4), (*P*_*median*_ = 0.015). Patients with ≥10 continuous medications had a significantly higher number of DRPs compared to those with < 10 continuous medications. In addition, patients with impaired renal function, GFR < 45 ml/min had a significantly higher number of DRPs compared to GFR ≥45 ml/min (Table 3).

**Table 3:** Patient data and DRP outcome according to number of medications and renal function

Patient data and DRP outcome according to renal function and number of medications	Total *n* = 109	< 10 continuous medications (*n* = 66)	≥10 continuous medications (*n* = 43)	*P*-value	GFR ≥45 ml/min *n* = 81	GFR < 45 ml/min *n* = 28	*P*-value
Age (years), median (range), mean	79 (52–98), 79	80 (58–98), 79	79 (52–91), 79	*P* = 0.816	77 (52–95), 77	84 (64–98), 83	***P*** **< 0.001**
Female, n (%)	60 (55.0)	36 (54.5)	24 (55.8)	*P* = 0.896	45 (56)	15 (54)	*P* = 0.856
GFR < 45 ml/min, n (%)	28 (25.7)	14 (21.2)	14 (32.5)	*P* = 0.185	–	–	
Number of medications per patient, mean (range)	12 (5–28)	9 (5–19)	15 (10–28)	***P*** **< 0.001**	12 (5–28)	13 (5–25)	*P* = 0.450
Number of continuous medications, mean (range)	9 (3–20)	7 (3–9)	13 (10–20)	***P*** **< 0.0001**	9 (3–20)	10 (3–19)	*P* = 0.232
Number of medications as needed, mean (range)	3 (0–11)	2 (0–11)	4 (0–10)	***P*** **= 0.002**	3 (0–11)	3 (0–10)	*P* = 0.723
Number of DRP, mean	3.9	2.9	5.3		3.4	5.1	
Number of DRP, median	4	3	5	***P*** **< 0.001**	3	5	***P*** **< 0.001**

**Fig. 3:**
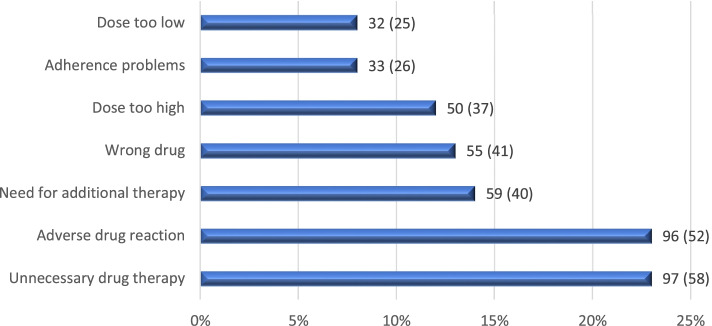
Distribution of the 420 identified DRPs by category, n = number of DRPs (number of unique patients)

The identified DRPs were classified into seven categories according to Cipolle, Strand, Morley et al. [1, 2], (Fig. 3). The most frequent categories were *Unnecessary drug therapy* and *Adverse drug reaction,* which represented 23.0 and 22.9% from the total amount of DRP categorized. One patient may have had more than one DRP in each category.

The most frequent medication groups involved in the DRPs, categorized according to the ATC-system, were C03; diuretics (9.6%), N05; psycholeptics - consisting of hypnotics and sedatives, anxiolytics, and antipsychotics (7.9%), R03; drugs for obstructive airways (7.7%) and N06; psychoanaleptics (7.7%). A total of 481 medications were involved in the DRPs as more than one medication could be involved in a DRP. Within the DRP category *Unnecessary drug therapy* antianemic preparations, mineral supplements, and diuretics were the most common medication groups, and in the category *Adverse drug reaction* diuretics, antithrombotic agents and psychoanaleptics appeared most frequently. However, a vast number of medication groups were involved in all DRP categories.

## Discussion

This study on pharmacist-led MRs for community-dwelling patients, whereof more than three-quarters were responsible for their own medication, shows that a large amount of DRPs were identified, thus strengthening the need for MRs for these patients. As in other studies, most of the patients were elderly individuals with polypharmacy [4, 23]. Up to one-third of the patients had impaired renal function in need of special consideration regarding medication treatment. The most frequent DRPs were *Adverse drug reaction* and *Unnecessary medication*.

On average 3.9 DRPs per patient were identified and most of the patients were exposed to at least one DRP. This is more than in several other studies (2.2–2.5 DRPs per patient) [14, 19, 20]. In an Australian study with a comparable selection of patients, 2.3 DRPs per patient were noted [14]. Two Swedish studies on MRs in nursing home residents found fewer DRPs per patient [19, 20]. However, in these studies, patients had round-the-clock nursing. Hence, the results from this study might indicate a great need for MRs for community-dwelling patients where risk situations are perhaps unidentified. Furthermore, the patients in our study were slightly younger than in previous studies [19, 20] but had an equivalent number of medications, which emphasizes a need to prioritize community-dwelling patients for MRs. The higher number of DRPs might also reflect the fact that the patients were specifically selected by the GP or nurse, which took the initiative for the MR, due to an expected need. This also indicates a high precision in selecting patients. A Canadian study identified 7.2 DRPs per patient [17], which is far more than most other studies. They only included patients with a recent loss of autonomy or a recent hospitalization with medication changes. This could partly explain their higher number of DRPs, due to more complex health situations, and the fact that the studied patient group had a higher number of medications on average. Polypharmacy as well as impaired renal function are known risk factors for DRPs [4, 24]. In our study patients with either impaired renal function or ≥ 10 medications had a higher number of DRPs, which might reflect an increased vulnerability and emphasize the need for MRs.

*Adverse drug reaction* and *Unnecessary drug therapy* were the most frequently identified DRP categories and represent a quarter each. The extent of the category *Adverse drug reaction* is larger than in previous Swedish studies conducted on patients living in nursing homes or independent living connected to municipal healthcare [19, 20]. The absence of healthcare personnel who normally identify and resolve adverse drug reactions could explain the higher number of this DRP category in our population. For patients living in nursing homes in Sweden, there is often a shift in medication treatment, from prevention to treatment of symptoms due to an expected limited lifetime. Therefore, it might be easier for the nursing home physician to deprescribe medications potentially associated with DRPs. That shift is not as clear for community-dwelling elderly patients; this might explain the higher rate of DRPs in our study. As in our study, Krska et al. identified suspected adverse drug reactions in a quarter of the studied patients when pharmacists identified pharmaceutical care issues in community-dwelling elderly individuals, aged 65 years or older, that were taking four medications or more on a regular basis [25].

Antithrombotic agents were one of the most frequent medication groups involved in the DRP *Adverse drug event*, as was also found in previous studies [26, 27]. According to Beijer et al. hospitalization caused by adverse drug reactions, due to, for example, antithrombotic agents, was more likely to be preventable in elderly patients [28]. They also suggested that pharmacists monitoring the medications of elderly patients may reduce these hospital admissions. This supports the importance of MRs for the target group in this study. A factor to consider is that medications with frequent use among the patients might have a higher occurrence among the identified DRPs. However, these DRPs are no less important to adjust.

*Unnecessary drug therapy* being at the top among our identified DRPs highlights the importance of structured MRs. Reducing unnecessary drug therapy could provide benefits for the patients both regarding less polypharmacy and lower risk of adverse drug reactions and drug interactions. On the other hand, *Unnecessary drug therapy* is lower in our study compared to previous studies in nursing homes (29–39%), which might be explained by the fact that community-dwelling patients could have better health and more to gain with medication treatment [19, 20]. This might also be reflected by the fact that the third most common DRP in this study was *Need for additional drug therapy*.

We found that the most frequent moderate or severe discomforts reported were being *tired/exhausted*, *poor sleep pattern/nightmares* and *dizzy/unsteady/high risk of falls*. It is yet to be determined if these symptoms are related to adverse drug reactions from current medication, for example nightmares from beta-blockers, exhaustion from antithrombotic-related anemia or dizziness from sedatives or antihypertensives.

The assessment tool, PHASE-20, does not include an overall question regarding pain, however *headache* and *abdominal pain/chest pain* are included, and “for example pain” is also mentioned in *other symptoms.* Despite this, almost three-quarters reported pain in some part of the assessment tool. Two thirds of the patients used N02; analgesics when needed or every day. These results show that many patients suffer from pain, thus indicating an important focus for MRs. Beuscart et al. emphasized in a previous study that the subject of pain was of great importance when patients rated relevant outcomes of MRs [29]. Under-treatment of pain is commonly noted in elderly patients, both community-living and nursing home residents [30, 31]. However, persistent pain medication is also noted as well as treatment with unclear indication (i.e. unnecessary drug treatment) [3, 32]. Hence individual regular follow-up and assessment are of high importance.

As much as one third of the patients were excluded. This was a retrospective study, with recruitment sometimes occurring a long time after the MR was conducted, which may explain the lapse. About two- thirds of the excluded patients were deceased, could not be reached, or could not give consent because of cognitive impairment. These patients might possibly have accepted to participate if recruitment was carried out closer to the intervention. The deceased group of patients might reflect a more frail part of this population and as their data are missing this might have biased the results. Another weakness in this study was that the participating pharmacists knew that their MRs were being studied, which might have affected their thoroughness and possibly resulted in a higher number of identified DRPs. However, the circumstances are often equal in similar studies.

A limitation is the heterogenicity concerning the estimation of GFR. Different methods were applied depending on accessible information in the EMR. All GFR estimations were based on creatinine but sometimes more extended laboratory tests including cystatin C could be used. Nevertheless, this reflects the reality for healthcare personnel.

In this study a modified version of LIMM has been used; an already well-established model that ensures the consistency in conducting MRs and facilitates the reproducibility. Participating clinical pharmacists had a master’s degree in clinical pharmacy or at least 4 years of experience working with clinical pharmacy. All were trained in conducting MRs, which was already a part of their customary work. Another strength of the study is the inclusion of various sites with differing socioeconomic areas and different types of PHCCs located in urban and rural areas reflecting a wide range of patients. In addition, with several participating GPs and clinical pharmacists, this indicates that the model is possible to practice in daily work. Thus, additional studies need to assess the participants’ opinions and experiences of the method to explore how the MRs can be further developed for this specific group. Future studies should also focus longitudinally to investigate how the results of the MRs are acted on by the GPs, if the DRPs decrease, and how this benefits the patients, for example, in terms of preventing hospital admissions and reducing mortality.

## Conclusion

A majority of the selected patients had at least one DRP, suggesting that selected primary healthcare patients in independent living benefit from an MR. Patients with impaired renal function or polypharmacy may need special attention. Further studies are needed to assess the participants’ experiences of the model.

## Supplementary Information

**Additional file 1.**

## Data Availability

The datasets used and analysed during the current study are available from the corresponding author on reasonable request.

## Abbreviations

ATC: Anatomical Therapeutic Chemical Classification System
CEPN: Swedish Central Ethical Review Board
COPD: Chronic Obstructive Pulmonary Disease
DRP: Drug-Related Problem
GP: General Practitioner
EMR: Electronic Medical Records
GFR: Glomerular Filtration Rate
LIMM: Lund Integrated Medicines Management
MR: Medication Review
PHASE-20: PHArmacotherapeutical Symtom Evaluation, 20 questions
PHCC: Primary HealthCare Center
TDM: Therapeutic Drug Monitoring
