# Supplementary material for: Pharmacist-led medication reviews in primary healthcare for adult community-dwelling patients – a descriptive study charting a new target group
Source: BMC Prim Care. 2022 Sep 16;23:237. doi: 10.1186/s12875-022-01849-x (PMC9482154; doi:10.1186/s12875-022-01849-x)
Supplement: Supplementary file 1 — Additional file 1. [file 12875_2022_1849_MOESM1_ESM.pdf]

PHASE-20 — rating scale for possible drug-related symptoms

Information about PHASE-20 is on the back of this form

Name	NHS number	N.I. number	Date

Document completed:

❑ Independently

❑ With support of relativ/next of kin/P.O.A

❑ With support of health professional Name:

Tick the box next to the option that best describes the level of discomfort during the last two weeks. Please leave clarifying comments in the margin when necessary. Circle the words that best match the symptoms and ~~cross out~~ those that do not match.

	No problem	Minor problem	Moderate problem	Severe problem	Comments
1. Dizzy/unsteady/high risk of falls	❑	❑	❑	❑	
2. Tired/exhausted	❑	❑	❑	❑	
3. Poor sleep pattern/nightmares	❑	❑	❑	❑	
4. Abdominal pain/chest pain	❑	❑	❑	❑	
5. Headache	❑	❑	❑	❑	
6. Low mood	❑	❑	❑	❑	
7. Worried/anxious	❑	❑	❑	❑	
8. Irritable	❑	❑	❑	❑	
9. Forgetful	❑	❑	❑	❑	
10. Poor appetite	❑	❑	❑	❑	
11. Dry mouth	❑	❑	❑	❑	
12. Nausea/vomiting	❑	❑	❑	❑	
13. Diarrhoea	❑	❑	❑	❑	
14. Constipation	❑	❑	❑	❑	
15. Palpitations (rapid/irregular heartbeat)	❑	❑	❑	❑	
16. Swollen legs/ankles	❑	❑	❑	❑	
17. Short of breath	❑	❑	❑	❑	
18. Frequent urination/incontinent of urine	❑	❑	❑	❑	
19. Itching/rash	❑	❑	❑	❑	

20. Other symptoms, which you have experienced during the past two weeks, (for example pain), indicate which.

❑ Minor problem ❑ Moderate problem ❑ Severe problem

Additional comments:

User-instructions for PHASE-20

The purpose of PHASE-20 (PHArmacotherapeutical Symptom Evaluation - 20 questions) scale is to assess if you experience symptoms that may be related to your medications. Your health practitioner greatly appreciates your help with this.

Tick the box that best describes the symptoms you have had over the **last two weeks**. Please use the comments box if you want to say something specific about a certain symptom.

Where more than one symptom is mentioned, for example dizzy/unsteady/high risk of falls, please circle the one that concerns you the most, or indicate that you are not affected by it by crossing it out.

Example:
If you feel very “unsteady” but not “dizzy”, write ~~dizzy~~/unsteady/high risk of falls

For relatives who may need to assist the completion of this document

Note that your relative should make the rating of the symptoms independently and without influence, as much as possible. Your task is primarily to help read and ask about each of the symptoms and complete the form. Keep in mind that it only concerns the last two weeks.

If you are assessing the symptoms as the representative of the patient, this should be noted on the form as it is important for the heath practitioner to know.

Mariann Hedström, Marianne Carlsson, Anna Ekman, Ulrika Gillespie, Christina Mörk & Kerstin Hulter Åsberg (2016): Development of the PHASE-Proxy scale for rating drug-related signs and symptoms in severe cognitive impairment, Aging & Mental Health, DOI: 10.1080/13607863.2016.1232364

For use outside Sweden, please contact
Mariann Hedström
PhD, Department of Public Health and Caring Sciences, Uppsala University, Sweden
E-mail: mariann.hedstrom@pubcare.uu.se
